# Acute Reciprocal Changes Distant from the Site of Spinal Osteotomies Affect Global Postoperative Alignment

**DOI:** 10.4061/2011/415946

**Published:** 2011-10-04

**Authors:** Eric Klineberg, Frank Schwab, Christopher Ames, Richard Hostin, Shay Bess, Justin S. Smith, Munish C. Gupta, Oheneba Boachie, Robert A. Hart, Behrooz A. Akbarnia, Douglas C. Burton, Virginie Lafage

**Affiliations:** ^1^Department of Orthopaedic Surgery, University of California-Davis, 3301 C Street, Suite 1500, Sacramento, CA 95817, USA; ^2^Department of Orthopaedic Surgery, New York University Hospital for Joint Diseases, 306E 15th Street, New York, NY 10003, USA; ^3^Department of Neurosurgery, University of California San Francisco, 400 Parnassus Street, San Francisco, CA 94143-0112, USA; ^4^Department of Orthopaedic Surgery, Baylor Scoliosis Center, 4708 Alliance Boulevard, Suite 810, Plano, TX 75093, USA; ^5^Department of Orthopaedic Surgery, Rocky Mountain Hospital for Children, 2055 High Street, Suite 130, Denver, CO 80205, USA; ^6^Department of Neurosurgery, University of Virginia, P.O. Box 800212, Charlottesville, VA 22908-0212, USA; ^7^Department of Orthopaedic Surgery, Hospital for Special Surgery, 535 E. 70th Street, New York, NY 10021, USA; ^8^Department of Orthopaedic Surgery, Oregon Health Sciences University, 3181 SW Sam Jackson Park Road, Portland, OR 97239, USA; ^9^San Diego Center for Spinal Disorders, 4130 La Jolla Village Dr., Suite 300, CA 92037-1481, USA; ^10^Department of Orthopaedic Surgery, University of Kansas Medical Center, 3901 Rainbow Boulevard, Kansas City, KS 66160-7387, USA

## Abstract

*Introduction*. Three-column vertebral resections are frequently applied to correct sagittal malalignment; their effects on distant unfused levels need to be understood. *Methods*. 134 consecutive adult PSO patients were included (29 thoracic, 105 lumbar). Radiographic analysis included pre- and postoperative regional curvatures and pelvic parameters, with paired independent *t*-tests to evaluate changes. *Results*. A thoracic osteotomy with limited fusion leads to a correction of the kyphosis and to a spontaneous decrease of the unfused lumbar lordosis (−8°). When the fusion was extended, the lumbar lordosis increased (+8°). A lumbar osteotomy with limited fusion leads to a correction of the lumbar lordosis and to a spontaneous increase of the unfused thoracic kyphosis (+13°). When the fusion was extended, the thoracic kyphosis increased by 6°. *Conclusion*. Data from this study suggest that lumbar and thoracic resection leads to reciprocal changes in unfused segments and requires consideration beyond focal corrections.

## 1. Introduction

Pedicle subtraction osteotomy (PSO) has increasingly become an accepted technique to correct spinal sagittal malalignment and relieve associated pain and disability. The indications, technique, outcomes, and perioperative complications for PSO have been well described [1–13]. An aspect concerning PSO technique that has not been well described, however, relates to global changes in spinopelvic alignment that occur following thoracic and lumbar PSO. It has been well demonstrated that osteotomy techniques produce a focal change in spinal alignment and can improve sagittal vertical alignment (SVA). However, there is little data on the postosteotomy behavior of the unfused spinal segments remote from the osteotomy site(s) and how the reciprocal changes that occur through these segments may affect global alignment. Kim et al. reported on results of 35 patients treated with lumbar PSO for sagittal malalignment at minimum 5-year followup [7]. In addition to reporting improved postoperative sagittal alignment, the authors noted that thoracic kyphosis (TK) increased from 22° to 31°, postoperatively. However, the upper instrumented vertebra (UIV) was T6 or cephalad in 20 of 35 patients and the average number of fused vertebrae was 11.7; therefore, the increase in kyphosis was likely a reflection of surgical technique and rod contouring rather than reciprocal changes occurring in segments cephalad to the osteotomy site. Similarly, Yang et al. noted an increase in postoperative TK following lumbar PSO and also noted an increase in postoperative lumbar lordosis (LL) following thoracic PSO; however, it was not indicated if these changes occurred within fused segments or occurred spontaneously [12]. Ikenaga et al. reported a greater incidence of late onset postoperative kyphosis progression among patients receiving shorter segment fusion following lower thoracic or lumbar PSO compared to longer segment fusion; however, the authors did not specifically evaluate the behavior of the unfused vertebral segments following PSO [14]. Jang et al. did demonstrate reciprocal changes through unfused vertebral segments following anterior and posterior spinal fusion (APSF) in the low lumbar spine for degenerative flat back syndrome and sagittal malalignment in 28 patients. The authors found that increased postoperative LL resulted in spontaneous increase in TK and sacral slope, concluding that anatomic thoracic and pelvic parameters normalize following surgical restoration of LL [15]. However, no patients in this series received corrective spinal osteotomy. 

Our hypothesis is that significant changes in focal alignment with a PSO will affect distal portions of the spine in a reciprocal manner, that is, after a lumbar PSO the lordosis will increase, and there will be a reciprocal increase in thoracic kyphosis and a decrease in pelvic tilt; conversely, a thoracic PSO will result in a decrease in thoracic kyphosis and decrease in lumbar lordosis. We further hypothesize that spontaneous reciprocal changes that occur in the unfused spine will be greater than the changes observed in surgically controlled fused segments, and that they will affect the final spinopelvic alignment. 

## 2. Methods

### 2.1. Patient Selection

This study is a consecutive multicenter retrospective case review of patients that underwent PSO surgeries and fusions either in the thoracic or lumbar spine. Data was extracted from an IRB-approved multi-center database involving 8 sites around the US. Inclusion criteria for this study included adult patients with spinal deformity and complete preoperative and postoperative sagittal full-length radiographs, and use of lumbar or thoracic PSO for deformity correction. Limited fusion was defined for the lumbar PSO as restricted to T10 and below while a limited thoracic fusion was limited to the L1 and above.

### 2.2. Data Collection

Office charts were reviewed at each site to collect demographic information (age, sex, weight, and height) as well as surgical summary. All patients had preoperative and postoperative (min 3 month FU) radiographic evaluation in a free-standing position [16]. Radiographic films were downloaded from PACS systems (Dicom format) or digitized through a Vidar scanner (Vidar Systems Corp, Herndon, Va, USA) with 75 dpi resolution and 12 gray levels.

### 2.3. Radiographic Measurements

**Figure 1:**
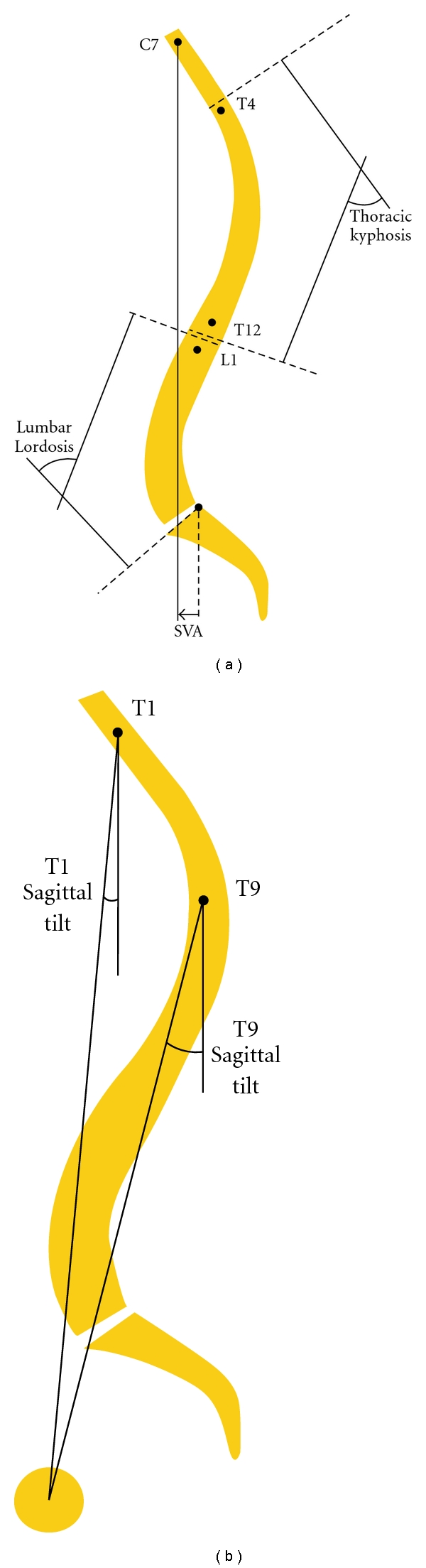
Sagittal spinal radiological parameters.

**Figure 2:**
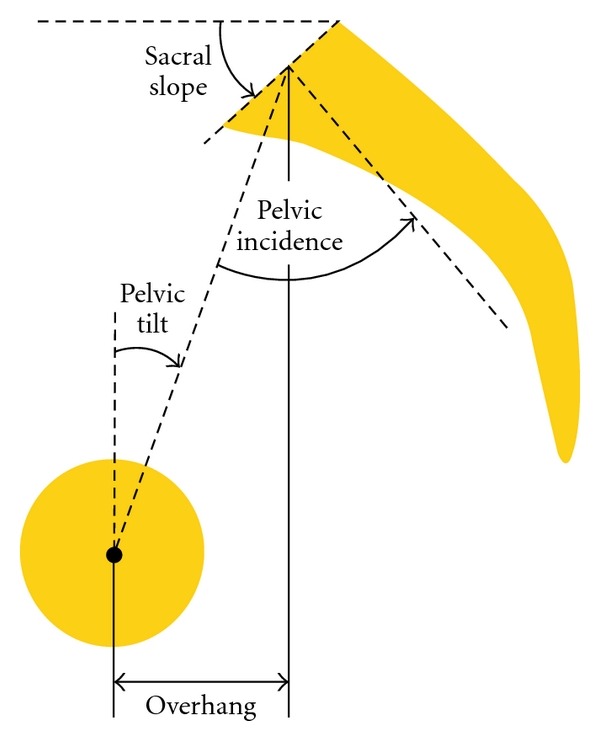
Pelvic parameters.

Preoperative and postoperative sagittal spinopelvic parameters were evaluated using Spineview software (Surgiview, Paris, France), a validated [17, 18] computer-based tool, which enables quantitative measurements of the spine and pelvis. The spinal sagittal plane (Figure 1) was described by calculating the L1-S1 lumbar lordosis, the T4–T12 thoracic kyphosis, the T10-L1 thoracolumbar kyphosis, the sagittal vertical axis (SVA), and the spinopelvic inclination of T1 and T9 (angle between the vertical plumb line and the line drawn from the vertebral body of T1 or T9 and the center of the bicoxofemoral axis). The sagittal pelvic morphology and orientation (Figure 2) were described by the pelvic tilt which is the angle between the vertical and the line through the midpoint of the sacral plate to the femoral heads axis, the sacral slope which is the angle between the horizontal and the sacral plate, and the pelvic incidence which is the angle between the perpendicular to the sacral plate at its midpoint and the line connecting this point to the femoral heads axis. PSO degree of resection (pedicle subtraction angle) was defined as the change of the angle formed by the lower vertebral endplate of the adjacent cephalic vertebra and the upper vertebral endplate of the adjacent caudal vertebra.

### 2.4. Statistical Analysis

Changes between preoperative and postoperative spinopelvic alignment were evaluated using a paired *t*-test analysis. Subgroup analysis was carried out for those patients that underwent a limited fusion after PSO correction. Differences among groups were analyzed using a one way ANOVA and unpaired *t*-test. The level of significance was set to 0.05. 

## 3. Results

### 3.1. Global Analysis

**Figure 3:**
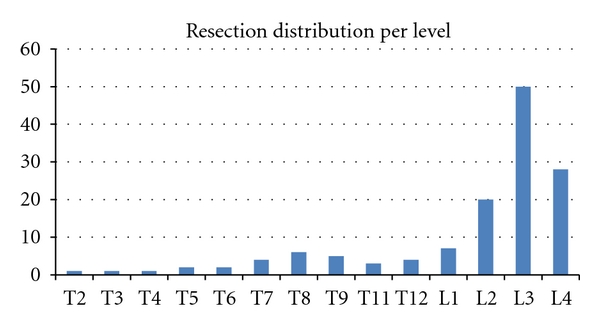
Resection distribution by vertebral level.

The review of the PSO database resulted in identifying a total of 134 consecutive adult patients (24 males and 110 females with a mean age of 54 years old SD = 12 years) with a mean BMI of 26.1 kg/m^2^ (SD = 5.1 kg/m^2^). These patients have a PSO for adult deformity, and the group consisted of 100 revision surgeries and 34 primary surgeries. Twenty-nine subjects underwent thoracic resection procedures (“Thoracic group”), and 105 underwent lumbar resection procedures (“Lumbar group”). Resection levels ranged from T2 to L4 (Figure 3), the majority of thoracic PSO corrections were accomplished in the T8 vertebral body, while the lumbar PSO corrections were most often performed in the L3 vertebral body. In the “*Thoracic group,*” the pre operative thoracic kyphosis of 53° was corrected to 38° (*P* < 0.001), the focal PSO resection was 11°, and the overall change in the lumbar lordosis was +8°. In the “*Lumbar group,*” the lumbar lordosis increased from 22° to 49° (*P* < 0.001), the average focal correction at the osteotomy site was 23°, and the overall change in thoracic kyphosis was +8°.

### 3.2. Subanalysis on the Limited Fusion Groups

These groups were subanalyzed, and 48 patients were found to have limited fusions, 12 patients in the thoracic PSO group and 34 in the lumbar PSO group. After a thoracic PSO, the thoracic kyphosis decreased from 66° to 38° (*P* = 0.001), and the unfused lumbar segment demonstrated a spontaneous decrease of lumbar lordosis from 70° to 62° (*P* < 0.05). After a lumbar PSO, the lumbar lordosis increased from 17° to 48° (*P* < 0.001), and the unfused thoracic segment demonstrated an increase in thoracic kyphosis from 22° to 35° postoperatively (*P* = 0.002). There was a normalization of the T1 spinopelvic inclination (from 5° to −3°, *P* < 0.001), preoperative SVA improved significantly from 143 mm to 44 mm post-op. (*P *< 0.001), and pelvic tilt improved significantly from 33° to 25° (*P* < 0.001).

### 3.3. Comparison between Long and Limited Fusion Groups

**Figure 4:**
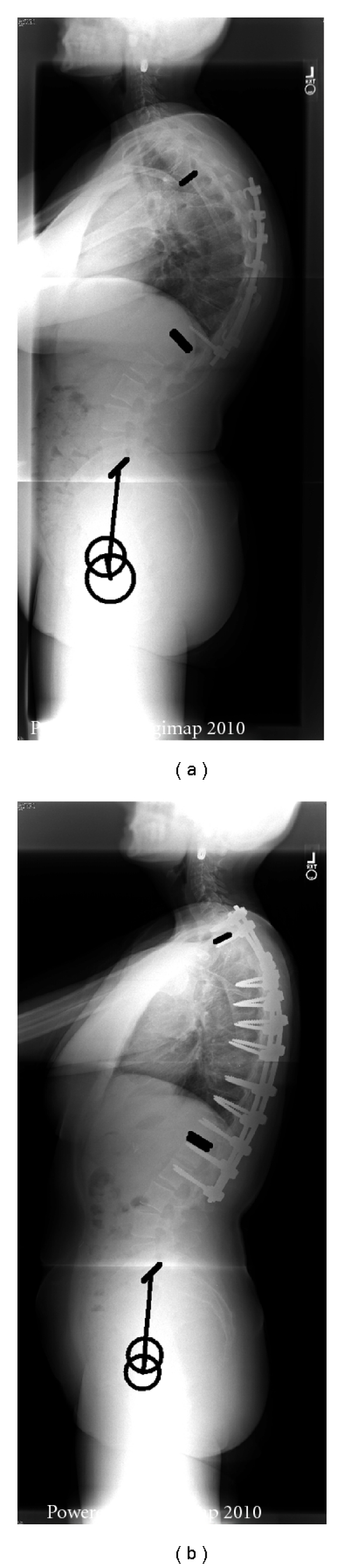
Thoracic resection, limited fusion.

**Figure 5:**
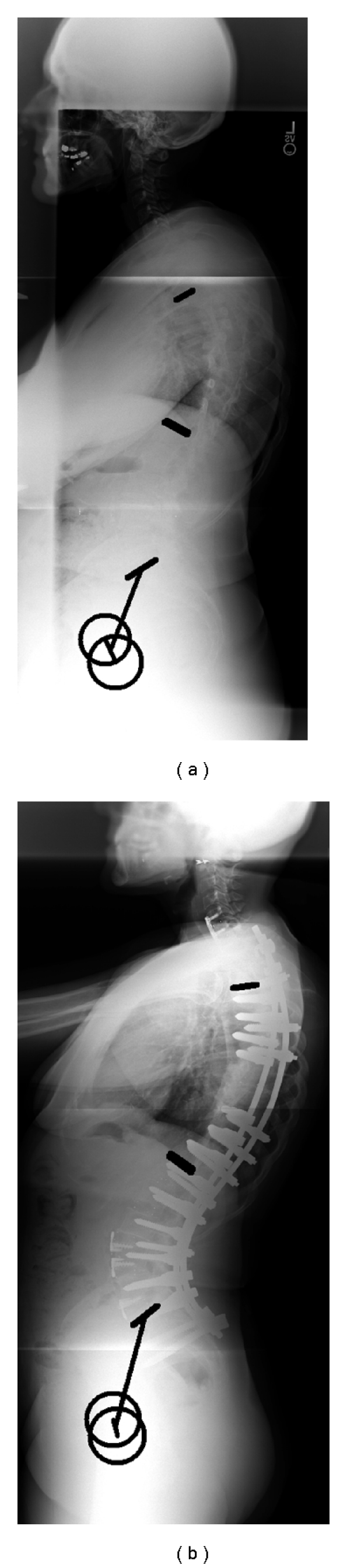
Thoracic resection, extended fusion to the lumbar spine.

In the *thoracic group *(Table 1), no significant differences were found between the long and the limited group in terms of demographic data (age, weight, and height). Patients with limited fusion presented with a smaller preoperative pelvic tilt than patients with long fusion (11° versus 25°, *P* < 0.001); they also presented with a larger preoperative lumbar lordosis (70° versus 52°, *P* = 008) while no significant difference was noted in terms of pelvic incidence. In other terms, patients with limited fusion had a preoperative lumbar lordosis larger than their pelvic incidence (+20°) while those with long fusion had a pre-op lumbar lordosis similar to their pelvic incidence. The PSO surgery led to a decrease of the lordosis in the limited thoracic fusion group (−8°) and an increase of the lordosis in the extended fusion group (+8°, *P* = 0.005). Both groups, therefore, exhibited a normalization of lumbar lordosis in comparison to their pelvic incidence. No significant changes were noted in terms of pelvic tilt for the limited fusion while the long fusion group had a decrease of the pelvic tilt (−8°, *P* < 0.001); postoperatively, the later group maintained a larger pelvic tilt than the limited fusion group (16.8° versus 9.6°, *P* = 0.028). Of note, no significant differences were observed in terms of pre- and postoperative SVA between the extended fusion group and the limited fusion group (*P *> 0.05). An example of patient with limited and extended fusion following thoracic resection is provided in Figures 4 and 5. 

**Figure 6:**
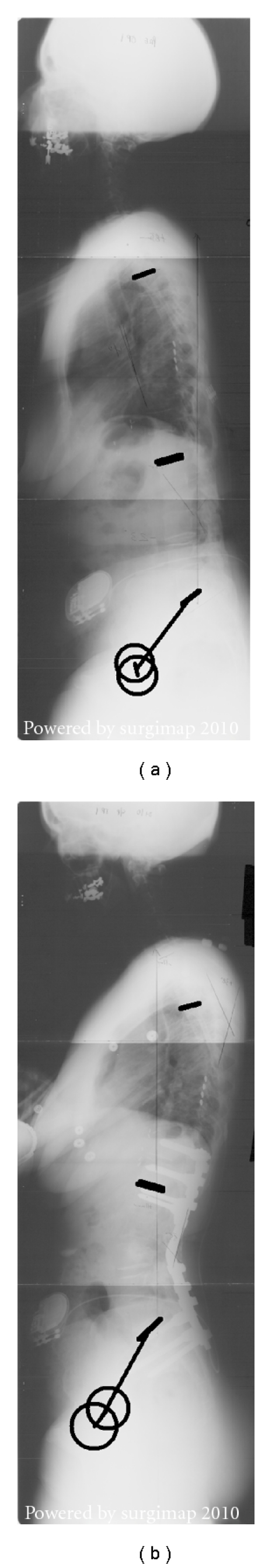
Lumbar resection, limited fusion.

**Figure 7:**
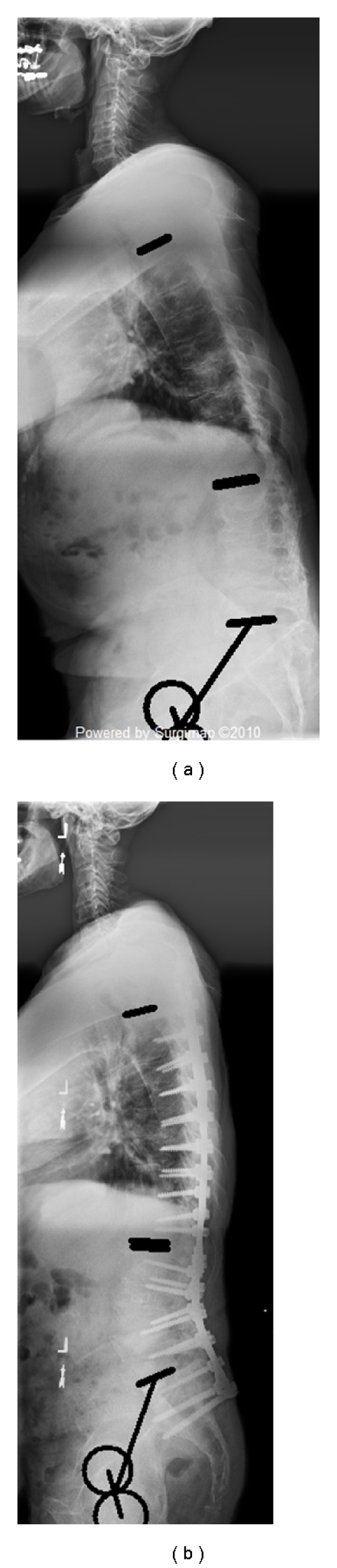
Lumbar resection, extended fusion to the thoracic spine.

In the *Lumbar Group *(Table 2), no significant differences were found between the long and the limited group in terms of demographic data (age, weight, and height). Patients with limited fusions presented with a smaller preoperative kyphosis than the long fusion group (22° versus 33°, *P* = 0.003) and did not have evidence of thoracolumbar kyphosis (2° versus 12°, *P* < 0.001). No other significant differences were noted preoperatively between the groups. The lumbar PSO surgery led to an increase of thoracic kyphosis in both groups although change in thoracic kyphosis was larger in the limited fusion group than in the long fusion group (+13° versus +6°, *P* = 0.004). Changes in lumbar lordosis and pelvic tilt were similar in both groups. Of note, no significant difference was observed in terms of pre- and postoperative SVA between the extended fusion group and the limited fusion group (*P *> 0.05). An example of patient with limited and extended fusion following lumbar resection is provided in Figures 6 and 7.

## 4. Discussion

Adult spinal deformity is a broad category that encompasses a diverse group of spinal malalignment patterns. It may range from a simple bi-planar deformity to more complex three- dimensional deformities with significant loss of coronal and sagittal alignment. For patients presenting significant rigid deformity, pedicle subtraction osteotomies can be utilized to create substantial changes in local and global alignment. This surgical technique requires multiple fixation points above and below the level of osteotomy. Extension of fusion beyond the adjacent levels is frequently required due to compensatory rigid deformity, additional deformity in adjacent regions, and the need to prevent adjacent level failure and further deformity. However, when possible, surgeons may attempt to correct the most rigid and deformed portions of the spine while leaving long segments unfused. This has the advantage of allowing continued flexibility and more normal motion in the nonfused portions. However, these unfused segments are dynamic and have the potential for ongoing changes in alignment to occur. 

In our retrospective series, the majority of surgeons chose to include distal or proximal fixation points in the thoracic or lumbar spine as part of the major deformity correction. These extended fusions commonly spanned from T4 to the pelvis (78% of the patients were fused to the sacrum). In the thoracic PSO group, 17 of 29 (59%) patients underwent a “long” fusion while 71 of 105 (68%) lumbar PSO patients had fusions that extended into the thoracic spine. This difference in preference for a limited fusion in the thoracic group is likely related to the surgical indication and pathology that was treated. Short-segment fixation may be acceptable for a PSO that plans on addressing a primary coronal curve with associated suboptimal sagittal alignment. The thoracic group was, of note, 10 years younger (mean age of 45 years old versus 55 years old, *P* < 0.001) than the lumbar group, which also may have influenced decision making to maximize the sparing of lumbar motion segments.

Patients who received selective thoracic fusions also tended to have a larger lumbar lordosis than those receiving long fusions (70° versus 52°). Conversely, the selective lumbar fusion group had a lower preoperative thoracic kyphosis, 22° versus 33°. It appears that in the lumbar fusion group long fusions were selected for patients with more substantial sagittal plane mismatch between the thoracic and lumbar spines: lower lumbar lordosis and greater thoracic kyphosis. Long fusions were also selected in patients with thoracolumbar kyphosis, most likely to avoid accelerated junctional failure. 

Surgeons commonly choose to perform longer fusions in the setting of adult spinal deformity in an effort to prevent accelerated degeneration, deformity, or kyphosis of the adjacent spinal segments and regions. Proximal junctional failure after lumbar PSO is a well-recognized entity, while distal junctional failure after thoracic PSO is less well understood [19]. Most of our literature regarding distal fixation points is derived from the Schueurmann's kyphosis literature and recommends that the distal extent of the fusion extends to the last lordotic disc [20–22]. Additionally, while avoiding extended lumbar fusions is felt to be critical for normal motion and to prevent adjacent level disease, extending fusions up into the thoracic region does not seem to elicit the same concerns of functional limitations or accelerated degeneration. 

Interestingly, the findings in this study demonstrate that fusions extended into the upper thoracic spine after lumbar PSO, only increased thoracic kyphosis minimally (+6°). However, in limited lumbar fusions, the unfused thoracic region demonstrated mean reciprocal changes of increased thoracic kyphosis by 13° (significantly greater than in long fusion group, *P* = 0.002). This reciprocal correction resulted in improved regional sagittal alignment (thoracic kyphosis post-op. mean 35°) and did not deter from the improved global alignment (SVA from 14 cm to 4 cm post-op.) and pelvic version (PT 32° to 25° post-op.). 

Following thoracic PSO, when the fusion extended into the lumbar spine, there was a mean increase in lumbar lordosis (+8°). On the other hand, when the lumbar spine was left unfused, the spontaneous reciprocal change led to a decrease of the lumbar lordosis 70° to 62° (*P* < 0.05). These changes allow a normalization of the lumbar lordosis in regard to the pelvic incidence.

The etiology of the reciprocal change is likely multifactorial. The preoperative spinal alignment represents the efforts of a maximally compensated spine, despite global malalignment. The compensatory portions of the thoracic or lumbar spine act to maximally increase or decrease their curvature to allow the head to be centered over the pelvis. It appears that with the correction of abnormal regional alignment in one portion of the spine, the unfused regions accommodate by “relaxing” the compensatory preoperative alignment to a more normalized one. The reciprocal changes may also reflect the central nervous system using this portion of the spine to once again center the head over the pelvis. The necessary degree of kyphosis or lordosis for optimal global spinal alignment can thus be achieved.

Kim et al. evaluated parameters that predicted optimal lumbar lordosis and sagittal alignment in adult spinal deformity (ASD) patients fused from the thoracolumbar spine (T9-L2) to L5 or S1 [23]. Patients with postoperative lumbar lordosis that exceeded thoracic kyphosis by 20°or more demonstrated optimal sagittal balance (defined as C7 plumb line falling within 3 cm of the posterior aspect of the L5-S1 disc) at minimum two-year followup. Patients with optimum sagittal balance, in turn, demonstrated superior health-related quality of life (HRQL) scores compared to patients with C7 plumb line greater than 3 cm from the posterior L5-S1 disc. However, all patients demonstrated postoperative progression of thoracic kyphosis over time. Kyphosis progression was similar between the optimal group and suboptimal groups; however, the suboptimal group had lower final postoperative lumbar lordosis and a greater percentage of patients with UIV ending in the lumbar spine, and, therefore, less ability to control reciprocal changes in the cephalad segments, compared to the optimal group. This phenomenon was recognized by Rose et al. who found that thoracic kyphosis did not change from preoperative to postoperative in patients fused to T5 or cephalad after lumbar PSO; however, patients fused caudal to T5 demonstrated significantly increased postoperative thoracic kyphosis (reciprocal change) [9]. The authors recommended the formula: lumbar lordosis ≤ 45°, thoracic kyphosis, pelvic incidence to predict optimal lumbar lordosis and sagittal alignment needed for lumbar PSO. However for selective fusion, reciprocal changes do occur, and thoracic kyphosis is not a static measurement. Consequently, formulas that do not appreciate this reciprocal change can underestimate the amount of PSO required to provide appropriate global sagittal alignment. 

This is a retrospective review of PSO's that were performed at multiple medical centers, which is an inherent limitation of the study. Variations in the specific technique of PSO as well as surgical indications are not accounted for. An additional limitation is the short followup for these patients with postoperative radiographs measured at three months. Further investigation into detailed analyses of reciprocal change patterns and evolution over time will be undertaken.

## 5. Conclusion

In an attempt to correct spinal malalignment, several key parameters are considered in preoperative planning. Although basic principles of spinopelvic alignment have been outlined, exact anticipation of postoperative alignment following correction osteotomies remains imprecise. From the findings in this study, it is evident that regional fusions with three column osteotomies can offer dramatic corrections. However, reciprocal changes require consideration beyond focal corrections in short segment fusions. In order to enhance surgical planning, and in order to avoid post operative alignment failures, greater appreciations of reciprocal changes are necessary. Accounting for these changes may prevent postoperative malalignment in some cases and permit greater confidence in pursuing selective fusions for select patients.

## Figures and Tables

**Table 1:** Comparison thoracic group—patients that had thoracic PSO. Preoperative and postoperative measurements, *P *< 0.05 in bold.

Thoracic group	Pre-Op			Post-Op			Change		
	Long fusion	Short fusion	*t*-test	Long fusion	Short fusion	*t*-test	Long fusion	Short fusion	*t*-test
T4T12 kyphosis (°)	53 ± 24	66 ± 28	0.088	38 ± 12	38 ± 12	0.457	−15 ± 25	−28 ± 25	0.089
L1S1 lordosis (°)	52 ± 18	70 ± 19	**0.008**	60 ± 12	62 ± 16	0.341	8 ± 19	−8 ± 7	**0.005**
T10-L1 kyphosis (°)	19 ± 20	10 ± 26	0.137	7 ± 8	8 ± 12	0.483	−12 ± 18	−2 ± 17	0.078
SVA (mm)	41 ± 92	−4 ± 59	0.076	−7 ± 56	−14 ± 61	0.390	−49 ± 71	−11 ± 23	0.051
T1 inclination (°)	−4 ± 10	−6 ± 4	0.300	−6 ± 5	−5 ± 4	0.234	−3 ± 8	1 ± 3	0.097
Pelvic tilt (°)	25 ± 10	11 ± 9	**0.000**	17 ± 10	10 ± 10	**0.028**	−8 ± 6	−2 ± 4	**0.003**
Pelvic incidence (°)	55 ± 10	50 ± 8	0.099	55 ± 10	50 ± 8	0.093	0 ± 2	0 ± 1	0.476

**Table 2:** Comparison lumbar group—patients that had lumbar PSO. Preoperative and postoperative measurements, *P *< 0.05 in bold.

Lumbar group	Pre-Op			Post-Op			Change		
	Long fusion	Short fusion	*t*-test	Long fusion	Short fusion	*t*-test	Long fusion	Short fusion	*t*-test
T4T12 kyphosis (°)	33 ± 16	22 ± 23	**0.003**	39 ± 15	35 ± 20	0.105	6 ± 11	13 ± 13	**0.004**
L1S1 lordosis (°)	22 ± 18	17 ± 21	0.144	49 ± 14	48 ± 16	0.371	28 ± 18	31 ± 17	0.187
T10-L1 kyphosis (°)	12 ± 13	2 ± 14	**0.000**	8 ± 10	7 ± 11	0.420	−4 ± 10	6 ± 10	**0.000**
SVA (mm)	142 ± 87	143 ± 73	0.489	45 ± 62	44 ± 47	0.469	−96 ± 67	−100 ± 59	0.399
T1 inclination (°)	5 ± 8	5 ± 6	0.494	−3 ± 6	−3 ± 4	0.399	−7 ± 7	−8 ± 5	0.383
Pelvic tilt (°)	34 ± 12	32 ± 11	0.198	26 ± 12	25 ± 10	0.320	−9 ± 10	−8 ± 8	0.321
Pelvic incidence (°)	58 ± 14	58 ± 12	0.452	58 ± 14	58 ± 13	0.422	0 ± 2	0 ± 3	0.304
